# Recent Studies on Cyclic 1,7-Diarylheptanoids: Their Isolation, Structures, Biological Activities, and Chemical Synthesis

**DOI:** 10.3390/molecules23123107

**Published:** 2018-11-27

**Authors:** Yurngdong Jahng, Jae Gyu Park

**Affiliations:** 1 College of Pharmacy, Yeungnam University, Gyeongsan 38541, Korea; 2 Advanced Bio Convergence Center, Pohang Technopark Foundation, Pohang 37668, Korea; jaegpark@gmail.com

**Keywords:** diarylheptanoid, biphenyl heptanoid, diphenyl ether heptanoid, total synthesis

## Abstract

Diarylheptanoids are a family of plant secondary metabolites with a 7 carbon skeleton possessing two phenyl rings at the 1- and 7-positions. They can be subdivided into acyclic and cyclic diarylheptanoids where the latter are further divided into *meta*,*meta*-bridged biphenyls ([7.0]metacyclophanes) and *meta*,*para*-bridged diphenyl ether heptanoids (oxa[7.1]metapara-cyclophanes). Since the isolation of curcumin from the rhizomes of turmeric (*Curcuma longa*) in 1815 which was named curcumin, a variety of diarylheptanoids have been isolated from a number of plant families such as Aceraceae, Actinidiaceae, Betulaceae, Burseraceae, Casuarinaceae, Juglandaceae, Leguminosae, Myricaceae, and Zingiberaceae. Earlier studies on these diarylheptanoids have been summarized on several occasions, of which the main themes only focus on isolation, structure elucidation, and the biological properties of linear types. Only a few have covered cyclic diarylheptanoids and their chemical synthesis has been covered lastly by Zhu et al. in 2000. The present paper has, therefore, covered recent progress in cyclic diarylheptanoids focusing on the isolation, structural and biological features, and chemical synthesis.

## 1. Introduction

Diarylheptanoids are a family of plant secondary metabolites with a seven-carbon skeleton possessing two phenyl rings at positions 1 and 7. They can be subdivided into acyclic diaryheptanoids (type **I**, linear) and cyclic diarylheptanoids where the latter are further divided into *meta,meta*-bridged biphenyls (type **II**, [7.0]metacyclophanes) [1] and *meta*,*para*-bridged diphenyl ether heptanoids (type **III**, oxa[7.1]metaparacyclophanes) [2,3].


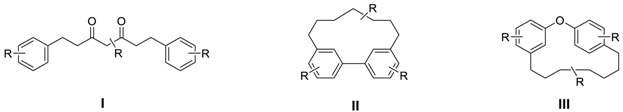


Vogel and Pelletier isolated a “yellow coloring-matter” from the rhizomes of turmeric (*Curcuma longa*) in 1815 and named it curcumin [4], and a century passed before the structure of curcumin by synthesis was defined as the first diarylheptanoid [5]. Since then, a variety of diarylheptanoids have been isolated from a number of plant families including Aceraceae, Actinidiaceae, Betulaceae, Burseraceae, Casuarinaceae, Juglandaceae, Leguminosae, Myricaceae, and Zingiberaceae. The extracts of these plants has long been used in traditional oriental medicine in China, India, Japan and Korea as well as ethno-medicine in western countries. Occurrences of three major diarylheptanoids in plants reported so far in the literature are summarized in Table 1. It should be noted that the Zingiberaceae family has long been the sources of many linear diarylheptanoids including curcumin; no cyclic diarylheptanoids, to the best of our knowledge, have been isolated as yet [6].

Their unique and characteristic structural features and wide range of biological properties have contributed to their popularity, which has led to continual isolation, evaluation of biological properties, and total synthesis. The earlier studies on diarylheptanoids were summarized by Claeson et al. [32,33], and by Lv and She [34,35], which included all of these three subcategories. Although the two reviews by Claeson et al. covered the general aspects of diarylheptanoids, the reviews by Lv and She focused on their structures, distributions, biological activities and ^13^C-nuclear magnetic resonance (NMR) spectral data of over 400 diarylheptanoids. In addition, additional reviews and papers covering linear diarylheptanoids have appeared recently [36,37,38,39], but reviews addressing cyclic diarylheptanoids are very limited. Furthermore, the main themes of the reviews focused on the isolation, structure elucidation, and biological properties while attention to synthesis was somewhat out of scope. Reviews by Keserü and Nógrádi [2] and by Zhu et al. [3] paid attention to the total synthesis of three categories of diarylheptanoids; no additional summaries have been made since then. The present paper has, therefore, covered the recent progress in macrocyclic diarylheptanoids with a focus on their isolation, structural features, biological properties, and synthesis.

## 2. Isolation, Structural Features, and Biological Properties

Diarylheptanoids have been typically isolated from the (inner) stem and the root bark of parent trees and shrubs, from the rhizomes of herbs, and also from the extracts of leaves and twigs of terrestrial plants. Two unusual sources of the green pericarps of walnuts, *Juglans regia* L. [40], and the nest of the paper wasp (*Polistes*), *Nidus vespae* [41], have been reported as possible sources of two types of cyclic diarylheptanoids; juglanin A (jugcathanin, **26f**) and B (**12ba**) from the former, and alnusone (**4a**) and its five congeners from the latter, respectively. Recently, the marine sponge *Tedania ignis* was found to be a new source of cyclic diarylheptanoids, tedarene A (**41a**) and B (**22**) [42].

Owing to the phenolic nature of diarylheptanoids, MeOH, MeOH/CH_2_Cl_2_ mixtures, and EtOH are commonly used for their extraction. However, acetone [43], hexane [44], and toluene [45] have also been used. A recent review paper by Alberti et al. summarized not only the methods and techniques of extraction and isolation, separation and characterization, but also the spectroscopic tools and techniques for structure elucidation of diarylheptanoids in detail [46].

### 2.1. Biphenyl Diarylheptanoids

Biphenyl heptanoids were classified into 4 categories based on their basic structural properties: (1) asadanin and related derivatives, (2) myricanone, myricanol, and related derivatives, (3) garuganins, and (4) miscellaneous.

#### 2.1.1. Asadanin and Related Derivatives

Asadanin (**1a**) was isolated from the MeOH extract of *Ostrya japonica* Sarg. (Betulaceae) by Yasue et al. as the first biphenyl heptanoid [14]. They deduced the structure by a series of elegant chemical conversion and degradation study, and later confirmed the structure by NMR spectroscopy [47]. Additional biphenyl heptanoids, such as deoxoasadanin (**1b**), epiasadanol (**1c**), isoasadanol (**2**), and di- and trideoxyasadanin-8-ene (**3**) were isolated from *Ostrya japonica* [48]. Recently, Singldinger et al. isolated asadanin (**1a**) from hazelnuts (*Corylus avellana* L.) as the main contributor to the bitter off-taste and reported complete analysis and assignment of all the proton and carbon resonances of the molecule [49].


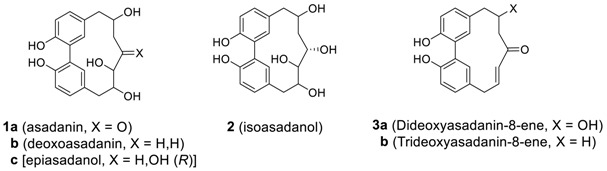


Nomura and Tokoroyama reported a series of related cyclic biphenyl diarylheptanoids, alnusone (**4a**), alnusonol (**5a**), and alnusoxide (**6**) from *Alnus japonica* (Betulaceae) [50]. Later, an alnusdiol (**7a**) was additionally isolated from the same source [51]. It should be noted that the absolute configuration of alnusdiol (**7a**) upon biphenyl axis was determined by X-ray and CD analysis to be a*S* and two additional chiral centers in the heptane skeleton were determined to be 3*S*,5*S* [52]. Alnusone and two other related compounds **4b**,**c** were also isolated from *Corylus sieboldiana* [53]. The same group isolated three more related heptanoids **5a**, **8**, and **9** from *C. sieboldiana* [54]. The isolation of diarylheptanoid **8** is somewhat surprising due to the presence of the isopropylidene group as a partial structure at the side chain even though its precursor **5c** has been isolated at the same time. Recently, the related compound **5d** (see Table 2), which was also isolated from *Alnus japonica*, showed very potent anti-adipogenic activity [55]. The diarylheptanoid alnusdiol (**7a**) was also isolated from *Casuarina junghuhniana* (Casuarinaceae), along with casuarinondiol (**5b**) [20] and *Betula maximowicziana*, along with **5b** and an alnusdiol glycoside (**7b**) [56] later. The casuarinondiol (**5b**) was additionally isolated from *Scyphiphora hydrolphyllaceae* (Rubiaceae) [30].


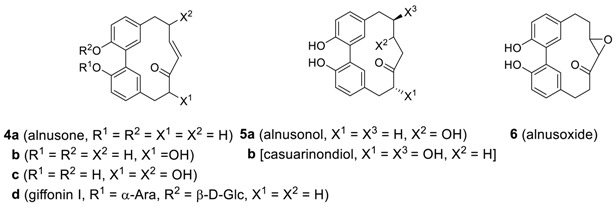



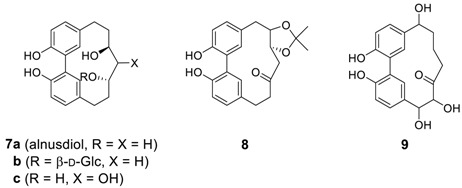


A series of the related derivatives of alnusonol (**5a**) and alnusdiol (**7a**) are summarized in Table 2. It should be noted that the biphenyl heptanoid **5j** has a unique substitution pattern on the biphenyl ring and has never been isolated elsewhere.

Structurally related systems have been isolated and named as aceroside XI, acerogenins E and K, and giffonins. Diarylheptanoid **5k** was isolated from *Acer nikoense* (Aceraceae) and named as aceroside XI [57], of which hydrolysis afforded the known heptanoid, acerogenin E **(5l**), the first synthetic biphenyl heptanoid previously reported from the hydrogenation of alnusone (**4a**) [50] and later isolated from the inner bark of *Betula ermanii* [58], along with acerogenin K (**7h**) [59]. Acerogenin E was also isolated from *Betula platyphylla* var. *japonica* [60] and *Acer nikoense* [59]. There are 11 species of Betula in Japan, in which the constituents vary; acerogenin E derivatives **10a**,**b** as the major biphenyl heptanoids in *B. davurica* [61]. Compound **10b** was also isolated from leaves of *B. platyphylla* [62] and showed promising leishmanicidal activity (IC_50_ = 28 μg/mL) [63]. In addition, the biphenyl heptanoid **10c** was isolated from the leaves and fruits of *Rhoiptelea chiliantha* (Rhoipteleaceae) [26].


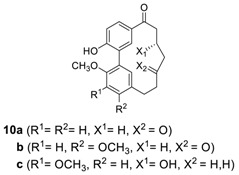


Recently, two series of biphenyl giffonins M, N, and T (**5e**–**g**) and giffonins L, O, P, and U (**7d**–**g**) were isolated from hazelnuts (*Corylus avellana*) [11,12] and are summarized in Table 2. The relative configurations of giffonins were established by a combined quantum mechanical (QM)/NMR approach, comparing the experimental ^13^C/^1^H NMR chemical shift and the related predicted values. Carpinontriol B (**5i**) and giffonin U (**7g**) at 40 μg/disk caused the formation of zones of inhibition against not only Gram-positive bacteria such as *Bacillus cereus* and *Staphylococcus aureus* but also Gram-negative *Escherichia coli* and *Pseudomonas aeruginosa* [64].

#### 2.1.2. Myricanone, Myricanol and Related Derivatives

The second series of biphenyl diarylheptanoids, myricanone (**11a**) and myricanol (**12a**) were isolated from *Myrica nagi* (Myricaceae) [68,69,70]. Myricanone has a variety of biological activities, which have been summarized in Table 3, whereas myricanol has an anti-tau activity thus having anti-Alzheimer’s disease activity. It has been figure out that the *aS*,11*R* enantiomer [(−)-**12aa**-(a*S*)(11*R*), vide infra] is responsible for the majority of tau-lowering activity [71].


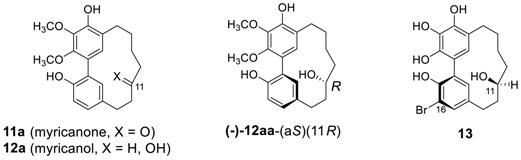


The absolute configuration at C-11 of myricanol (**12aa**) was determined by an X-ray crystal structure on 16-bromomyricanol (**13**), prepared by brominating myricanol, and found to be a*R* [69,70]. Although the absolute configuration of secondary alcohol at C-11 was assigned as *S*, the authors did not mention about the atropisomerism [72,73] of the molecule developed by biphenyl axis. However, the configuration of the biphenyl axis of 16-bromomyricanol could be readily assigned to *P* (more likely a*R*) configuration by carefully analyzing X-ray crystal structure. Myricanol has also been isolated from *M*. *gale* [74], *M. rubra* [28], *M. esculenta* [75], and *M. cerifera* [44], and *Morella salicifolia* [29] as well. Later, an extensive NMR study revised a previous ^13^C-assignment and X-ray analysis study of (±)-myricanol that confirmed the structure [44]. In addition, additional derivatives of myricanone and myricanol as well as their glycosides isolated so far reported, are summarized in Table 3 and Table 4.

It should be noted that a series of myricananins A–H (**12e**, **12f**, **11g**, **14**, **11h**, and **12g**–**i**) were also isolated from *Myrica nana* and showed inhibitory activity (IC_50_ = 45–63 μM) on nitric oxide release in LPS-activated peritoneal macrophages [82].

A compound with a close structural relationship to myricanol (**12a**) in structure, was isolated from *Juglans regia* and named as juglanin B (**12ba**) [40]; the structure of this was originally proposed in error [91]. Subsequently, two esters of sulfuric acid such as myricanol 11-sulfate (**12ab**) and juglanin B 11-sulfate (**12bb**), and related glycosides (**12j**–**n**) were isolated from *M. rubra* [89]. The juglanin B (**12ba**) showed cytotoxic activity against human hepatoma Hep G2 cells [40].


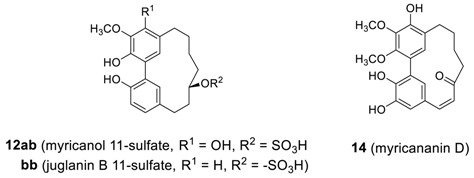


Four additional related heptanoids **15** were isolated from *Myrica gale* [92]. The heptanoid **15a**, known as porson, was first isolated from the same plant by Anthonsen et al. [74], whose structure proposed was revised 20 years later by Nagai et al. [92]. Saliciclaireone B (**16**), a regioisomer of **11d** (saliciclaireone A), was also isolated from *Morella salicifolia* [29]. Porson showed promising anti-tuberculosis activity, with an MIC value of 40 μg/mL [79].


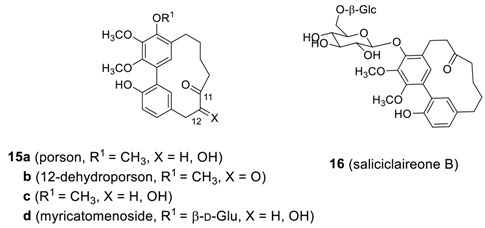


#### 2.1.3. Garuganins

A series of biphenyl heptanoids with a heptene skeleton have also been reported. Garuganin II (**17a**) [93], garuganin V (**17b**) [17], and 6′-hydroxygaruganin V (**18**) [18], were isolated from *Garuga pinnata* as biphenyl heptanoids in the garuganin series I-VII (*vide infra*). The X-ray structures of garuganin II [93] and V [94] showed that the (*Z*)-heptene is the basic skeleton, unlike the (*E*)-heptene of alnusone series. As it is thought that adapting (*E*)-alkene geometry to accommodate a bulky methoxy substituent inside the cyclic diarylheptanoids generally imposes substantial strain to the ring system, the (*E*)-heptene structure in 6′-hydroxygaruganin V (**18**), proposed by Ara et al. is somewhat surprising. Indeed, the minimized total energies of the two (*Z*)-**18** with (*E*)-hepta-6-en-5-one skeleton and (*E*)-**18** with (*Z*)-hepta-6-en-5-one skeleton calculated by MM2 are 166.8 Kcal/mole and 45.6 Kcal/mole, respectively, reflecting the fact that **18** with (*Z*)-heptene moiety is much more stable. The structure of **18**, thus, remained to be clarified. Garuganin V showed strong bactericidal activity against *Bacillus sereus* and *B. subtilus* as well as Gram-negative *Salmonella enterica paratyphi* [95].


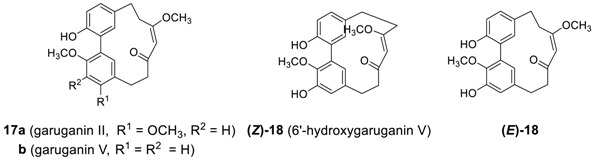


#### 2.1.4. Miscellaneous

The structurally novel diarylheptanoid actinidione (**19**) was isolated from the leaves and twigs of *Clematoclethra actinidioides* (Actinidiaceae) [8]. It is somewhat surprising that **19** is the first biphenyl heptanoids with a [7.0]orthometacyclophane skeleton and a 1,4-benzoquinone, and showed promising cytotoxicity (GI_50_ = 16.03–32.58 μg/mL) against Lun-06, Neu-04, Bre-04 cell lines. This compound was later isolated from the bark of *Myrica nana* [82] along with two additional new [7.0]metacyclophanes, rubanol (**20**) [96] and nanaone (**21**) [97], in which one benzene ring was oxidized to 1,4-benzoquinone. As one may expect from the 1,4-benzoquinone moiety, **19** showed strong antioxidant activity (IC_50_ = 7.9 μg/mL) against superoxide dismutase (SOD) [89].


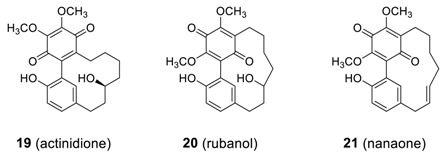


Recently, biphenyl heptanoid tedarene B (**22**) was isolated from marine sponge *Tedania ignis* [42]. Tedarene B has a unique structure with three chiral elements: one stereogenic carbon (C-9), one chiral axis through the biphenyl unit, and one chiral plane in the double bond, yielding 4 potential atropisomers for each of the two configurational stereoisomers at C-9; that is, 4 possible diastereomeric atropisomers of the configuration stereoisomer 9-*S*, i.e., (9*S*,2*S*_a_,10*S*_p_ or 9*S*,2*M*,10*M*), (9*S*,2*S*_a_,10*R*_p_ or 9*S*,2*M*,10*P*), (9*S*,2*R*_a_,10*S*_p_ or 9*S*,2*P*,10*M*), and (9*S*,2*R*_a_,10*R*_p_ or 9*S*,2*P*,10*P*) [98]. Among them, **22a** (9*S*,2*S*_a_,10*S*_p_) was isolated, but the ^1^H NMR showed a 4:1 equilibrium mixture of **22a** and **22b** after 24 h due to the conformational isomerism of the alkene moiety as shown below.


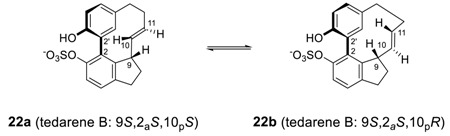


### 2.2. Diphenyl Ether Diarylheptanoids

The diphenyl ether diarylheptanoids were categorized into three major groups based on their skeletal properties: (1) acerogenins and acerosides, (2) garuganins and garugamblins, and (3) miscellaneous.

#### 2.2.1. Acerogenins and Acerosides

Nagai et al. reported the first diphenyl ether diarylheptanoid, acerogenin A (**23b**) [99] and its glycoside (**23a**) [100] from *Acer nickoense* and the latter could be enzymatically hydrolyzed to **23b**. Later its regioisomer, **24a**, was isolated from the same plant and named as acerogenin B (**24a**) [101]. One of the most impressive characteristics of **23b** and **24a** is the chemical shift of H^2^, which resonate at δ 5.84 and 5.46, respectively. Such resonances are highly upfield-shifted compared to the normal chemical resonances of protons (δ 7.26) on the benzene ring [102] and neighboring H^4^ (δ 6.63 for **23b** and 6.65 for **24a**) in the molecule. Such a shift of resonances can be explained by the fact that this proton orients towards the shielding region imposed by the ring current of the neighboring phenyl ring [103]. X-ray crystallographic analysis of the related system such as galeon (**26c**) [104], maximowicziol (**24h**) [52], and acerogenin B (**24a**) [105] has revealed that the main planes of the two phenyls are very close to 90^o^ to each other.


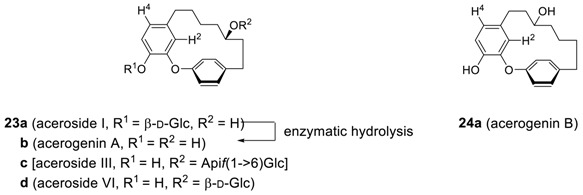


Continued studies on the same plants led to the isolation of an additional cyclic diaryl ether heptanoid, aceroside IV (**25a**) as a glycoside. Hydrolysis of glycoside **25a** afforded a new diarylheptanoid, acerogenin C (**25b**) [106], which was additionally isolated from *Boswellia ovalifoliolata* [19] later. It should be noted that a regioisomer of **25b** was also isolated from *Acer nikoense* and named as acerogenin L (**26a**) [64].


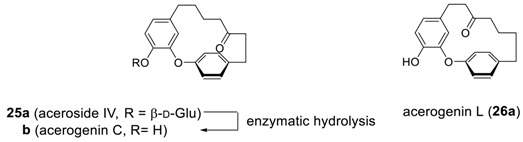


Additional series of cyclic diaryl ether heptanoids, isolated so far, and their biological properties are summarized in Table 5.

To determine the geometries of acerogenins, the splitting pattern of the 4 protons (H^2^, H^3^, H^5^ and H^6^) on *para*-disubstituted phenyl rings of acerogenins A (**23b**), C (**25b**), B (**24a**) and L (**26a**) in their ^1^H NMR spectra may afford important information. Only two resonances were found for these protons appearing as an AB quartet (^3^*J* = 8.3 Hz) in the spectra of acerogenins C and L, while four resonances, each being a doublet of doublet, were observed for the those of acerogenin A and B, respectively. These results may indicate that two sets of protons on the *para*-substituted phenyls in acerogenins A and B, such as H^2^ and H^6^, H^3^ and H^5^, are not equivalents, and may indicate a possible difference in the rotational energy barrier around the diaryl ether bond, unlike those in acerogenins C and L. The energy barriers in acerogenins C and L are, therefore, somewhat lower than those of acerogenins A and B, respectively. Indeed, the minimized energies of **25b** (2.65 Kcal/mole) and **26a** (3.85 Kcal/mole) calculated by Chem3D Pro^®^ are much lower than those of **23b** (10.11 Kcal/mole) and **24a** (10.15 Kcal/mole). The presence of *sp*^2^-hybridized carbon (carbonyl) in the heptyl chain of **25b** and **26a** may reduce both angle strain and H–H steric interactions relative to those of **23b** and **24a**, respectively. Such a rotational energy barrier in the systems with a substituent such as galeon would be increased sufficiently enough not to rotate, leading to two atropisomers. Indeed, galeon has been isolated on two separate occasions in both levo- [104] and dextrorotatory [112] ones from *Myrica gale*, respectively. The configuration of planar chirality was determined by X-ray crystallographic analysis of the corresponding 4-bromobenzoate (**26d**) of (−)-galeon to be *M*-(−)-galeon (**26cb**) and thus dextrorotatory one was determined as *P*-(+)-galeon (**26ca**). It should be noted that the nature of conformational chirality, the racemization mechanisms, and predictions of these for diaryl ether heptanoids have been recently studied [119].


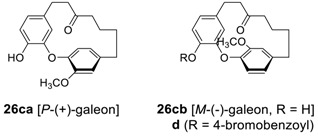


After we isolated galeon (**26**) [22] and its derivatives **27a**,**b**,**c** [23] from the roots of *Juglans mandshirica*, and a series of related diaryl ether heptanoids, **27d**-**f** from the stem of *Engelhardia roxburghiana* [21,120], were additionally isolated. Unfortunately, the plane chirality was not defined in all cases and remained to be clarified. The originally proposed structure **28** [120] as engelhardione was later revised as to **27f [21]**, which is identical to previously reported pterocarine (**26b**) [25] and revised structure was confirmed by total synthesis (vide infra). The compound **27c** showed strong inhibitory activity (95.7%) on topoisomerase II at 50 μg/mL level [121]. Engelhardiols A (**27d**) and B (**27e**) displayed anti-tubercular activity with MIC values of 72.7 and 62.1 μM, respectively [21]. Related engelhardione has MIC of 0.2 μg/mL against *Mycobacterium tuberculosis* (H37Rv) [120].


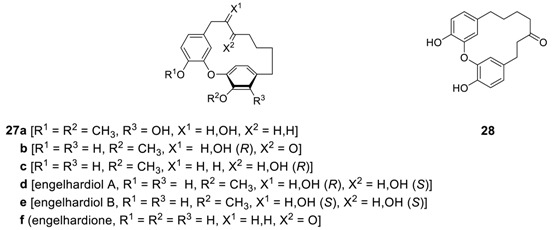


#### 2.2.2. Garuganins and Garugamblins

A series of cyclic diarylheptanoids with a double bond in a heptyl skeleton were isolated such as **29a** (garugamblin-1) and **31** (garugamblin-2) from *Garuga gamblei* [122,123], garugamblin-3 from *Alnus japonica* [124], and **29b** (garuganin I) [125,126], **30a** (garuganin III) [127], **30b** (garuganin IV), **32** (garuganin VI) [17], and related garugamblins such as **29c** and **30c** [18] from *Garuga pinata*. Although the structures of garugamblin-1 (**29a**), garugamblin-2 (**31**) [123] and garuganin I (**29b**) [125] were confirmed by X-ray structure analysis, the originally proposed structures of garuganin III (**30a**), garuganin IV (**30b**), and 1,9′-didesmethylgaruganin III (**30c**) were revised later through total synthesis to be **33**, **29a**, and **30d** (garuganin VII), respectively [128,129].


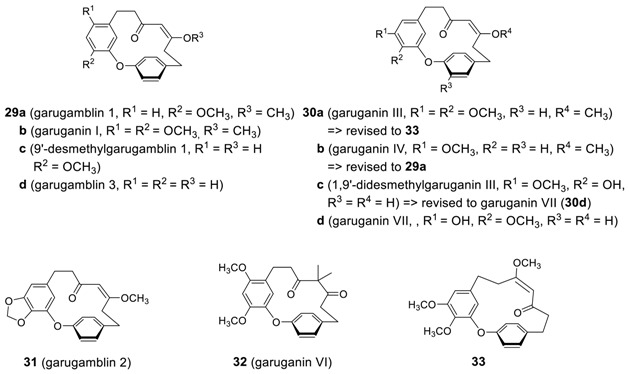


It should be noted that recent dynamic NMR studies revealed that garuganin I can undergo conformational enantiomerism, leading to racemization through the rotation of the C^7^–C^8^ and C^12^–C^13^ sigma bonds [130].


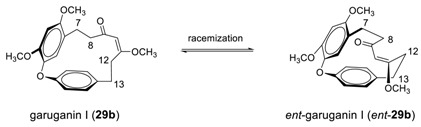


#### 2.2.3. Miscellaneous

In addition, a very potent (IC_50_ = 3.9 μg/mL) leishmanicidal [59] diaryl heptenoid, **34** from *Betula platyphylla* [65], *B. davurica* [66], and *B. ovalifolia* [131], and ovalifoliolatin (**35**) from *Boswellia ovalifoliolata* [19] were reported as additional diaryl ether heptenoids. The cyclic diaryl ether heptenoids **35** are unusual *trans*-cycloalkenes isolated from the nature and showed potent antibacterial activity against *Staphylococcus aureus* and *Chromobacterium violaceum*.


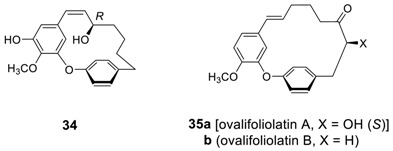


An additional series of diaryl ether heptenoids, giffonins A–K, Q, R, and S, were isolated from the leaves of the hazelnut tree (*Corylus avellana*) [11,12,132] which are summarized in Table 6. Diaryl ether heptenoids, giffonin D and giffonin H at 10 μM reduced both H_2_O_2_^−^ and H_2_O_2_/Fe^2+^-induced lipid peroxidation by more than 60% and 50%, respectively, being more active than curcumin (19.2%). When hazelnuts were infected by so-called ”Cimiciato”, the composition of the constituents was changed to produce a new oxa[7.1]metametacyclophane, 40, along with asadanin (**1a**) and giffonin P (**7f**) [133].


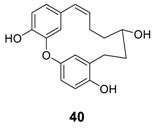


A unique cyclic diaryl heptadienoid tedarene A (**41a**) was isolated from the marine sponge *Tedania ignis* [42] and exhibited a couple of intriguing features. Although two double bonds can impose strain onto the cyclic heptanoid ring in tedarene A, the energy barrier between the two atropisomers is not sufficiently high to prevent interconversion at room temperature showing only one set of ^1^H resonances in NMR spectrum without any of protons on para-substituted phenyl ring. Such a phenomenon reflects that the interconversion rate between the two atropisomers is, coincidently, in the coalescence region at room temperature to cause the proton resonances of H^2^ and H^6^, as well as H^3^ and H^5^, of the *para*-substituted phenyl ring to be sufficiently broadened to be undetectable. The rotational energy barrier of **41a** was calculated by molecular dynamics simulation to be 14.0 Kcal/mol, which is way below the energy barrier (23.3 Kcal/mol) to define atropisomers [134,135]. A diphenyl ether heptatrienoid **42** was also isolated from *Ostryopsis nobilis* (Betulaceae) and the structure was confirmed by X-ray crystal structure analysis [16].


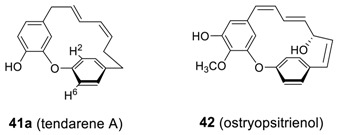


## 3. Total Synthesis of Biphenyl Diarylheptanoids

### 3.1. Biosynthetic Pathway

**Scheme 1:**
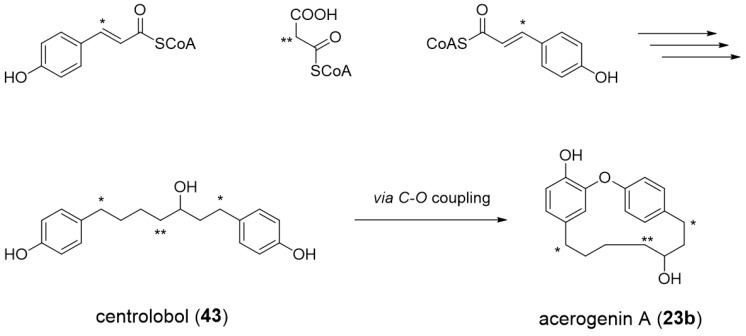
Propsed biosynthetic pathway for the linear and cyclic diaryl ether heptanoids.

Before the total synthesis of cyclic diarylheptanoids was considered, studies on the biosynthetic pathways are addressed. Elegant studies on the degradation of acerogenin A and the radioisotope-tracing biosynthetic pathway for 1,7-diaylheptanoids revealed that two cinnamate units coupled to a malonate via acetyl CoA leading to the 1,7-diphenylheptanoid, curcumin [136,137], which was further converted to centrolobol. In addition, Fujita and his coworkers performed two radioisotope experiments [138]: They fed labelled compounds, dl-[3-^14^C]phenylalanine and [3-^14^C]cinnamic acid to the young shoots of *Acer nikoense* and found that 95% of the radioactivity of acerogenin remained on the C1/C7, but in the case of l-1-^13^C]phenylalanine, only 5% of the radioactivity was incorporated at the positions C1/C7 in acerogenin A. On the other hand, feeding [2-^14^C] sodium acetate, [2-^14^C]malonic acid, and [1-^14^C] sodium acetate resulted in the sufficient incorporation of [2-^14^C] sodium acetate and [2-^14^C]malonic acid into the acerogenin, but not [1-^14^C] sodium acetate. These observations indicate the possible biosynthetic pathway of acerogenin A (Scheme 1), via an intramolecular oxidative coupling of its linear precursor, centrolobol (**43**).

It should be noted that Watanabe et al. isolated alnusonol (**5a**) along with its suspected precursor platyphyllonol (**44**) [53], from which one may deduce the presence of dicarbon radical as a possible intermediate in the biosynthetic pathway for the (9*S*)-alnusonol (**5a**) via C–C coupling.


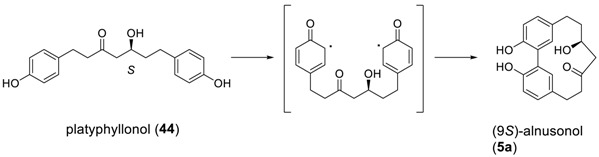


Such a premise was proved by the isolation of three cyclized diarylheptanoids such as acerogenin A (**23b**), (*R*)-acerogenin B (**24a**), and acerogenin E (**5l**), and a linear diarylheptanoid, (−)-centrolobol together from the same plants indicates that the former two are biosynthetically related to (−)-centrolobol [107]. In addition, many plants have afforded various combinations of two from the three diarylheptanoids (i.e., linear, biphenyl heptanoids and diphenyl ether heptanoids). One possible explanation for these results comes from the intramolecular oxidative coupling of phenolic linear diarylheptanoid via the free diradicals: A phenolic oxidative C–C coupling would lead to *meta*,*meta*-bridged biphenyl heptanoids, whereas the corresponding C–O coupling would result in the alternative, *meta*,*para*-bridged diphenyl ether heptanoids.


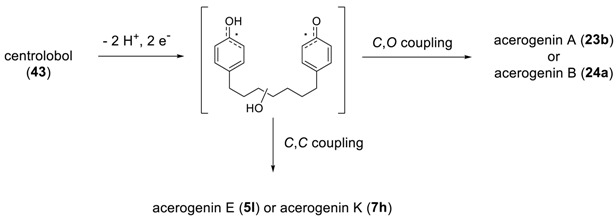


### 3.2. Total Synthesis of Biphenyl Heptanoids

The formation of the aryl–aryl bond has long been of interest in the development of facile synthetic methods, i.e., metal catalyzed coupling reactions, such as Suzuki [139], Stille [140], Negishi [141], Ullmann [142,143], and electro- as well as photochemical methods [144]. Among these, the Suzuki reaction, the Ullmann reaction, and the photochemical process have been applied to biphenyl diarylheptanoids at the final step. The Wittig [145], Thorpe [146], and olefin metathesis [147] reactions have also been employed for the C–C bond formation in the heptane moiety. However, attempts using Thorpe reaction failed to afford the desired cyclic biphenyl heptanoids [148], which would, thus, not be discussed herein.

#### 3.2.1. Aryl–Aryl Bond Formation via Metal Catalyzed Coupling

The Ni(0)-promoted intramolecular coupling of aryl halide developed by Semmelhack [149] was applied to the total synthesis upon biphenyl heptanoid, alnusone (**4a**) for the first time [150]. The reaction of diiodide **49** in the presence of tetrakis(triphenylphosphine)nickel afforded methyl-protected alnusone (**50**) in 46% yield. The acid-catalyzed deprotection of methyl group produced **4a** with 72% yield.


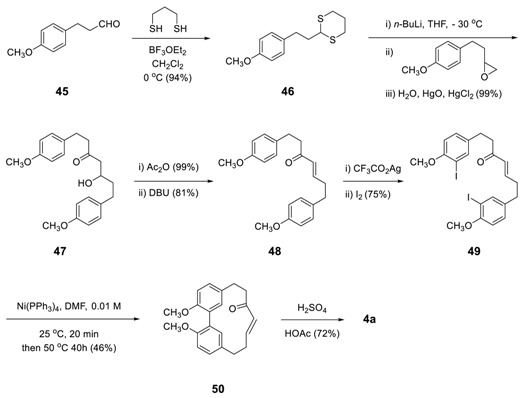


The starting compound **49** was prepared from 3-(4-methoxyphenyl)propanal (**45**) via thioacetal formation with propane-1,3-dithiol, lithiation by *n*-BuLi, a reaction with 2-(4-methoxyphenethyl)oxirane, dehydration by DBU, and iodination.

The same strategy has been applied to the synthesis of myricanone and myricanol by Whiting and Wood [151]. The intramolecular ring closure of diiodides **54** provided the corresponding benzyl-protected myricanone (**55a**) and myricanol (**55b**) in 10% and 7.3% yield, respectively. The low yield was explained by angle strain and van der Waals hydrogen interaction caused by the *sp*^3^-hybridized methylene units compared to those of *sp*^2^-hybridized *E*-methine in the former molecule, alnusone. The subsequent hydrogenolysis of **55a** afforded **11a** while **12a** was prepared by hydrogenolysis of **55b** followed by hydrolysis. It should be noted that authors did not mention about either the presence of stereoisomers or NMR spectral data for the compound **55b** even though **55b** has both a chirality center and a chiral axis. The starting **54a**,**b** were prepared by iodination of **53a**,**c**, respectively. The Grignard reagent generated from **51** was reacted with 3-(4-benzyloxyphenyl)propanal (**52**) to provide **53a** in 21% yield. Acetylation of **53a** gave **53b** and oxidation of **53a** with pyridinium chlorochromate (PCC) gave **53c** [152].


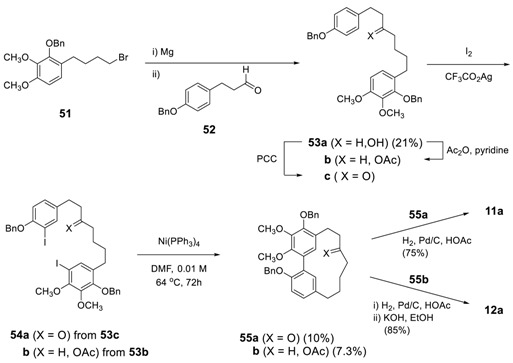


Ogura and Usuki employed the same strategy to prepare acerogenin E (**5l**) and K (**7h**) [153], in which the Ni(0)-catalyzed reaction was modified by a more recent methodology of a domino sequence of a Miyaura arylborylation and an intramolecular Suzuki reaction [154] of **59** to dimethylacerogenin E (**60**), of which deprotection by BBr_3_ to yield acerogenin E (**5l**). The reduction of **60** with NaBH_4_ was followed by demethylation with BBr_3_ to provide acerogenin K (**7h**) in 95% yield. The starting **59** was prepared from **56** via Claisen–Schmidt condensation, catalytic reduction, and CF_3_CO_2_Ag mediated iodination.


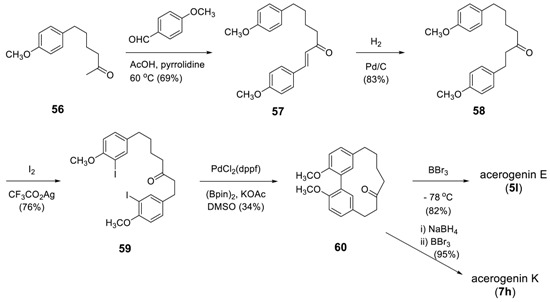


The similar oxidative homocoupling of the diboronic ester (**62**) also gave **60** in 50% yield [155]. The starting **62** was prepared from **58** via glycol protection of ketone, reaction with Cr(CO)_6_, borylation with isopropoxy-4,4-5,5-tetramethyl-1,3,2-dioxoborane, and the deprotection of ketone.


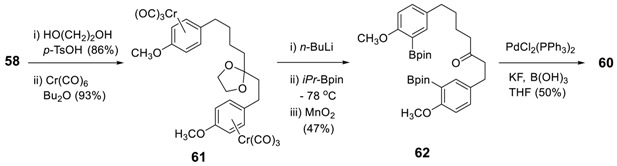


It should be noted that neither the reduction of the double bond in **63** to **64** nor the direct cyclization of **63** to **65** under Suzuki–Miyaura coupling was successful [153]; thus, the cyclization of **64** using the above reaction conditions has not been pursued.


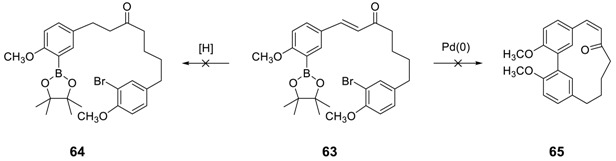


Such a method was revisited by Martin et al. for the synthesis of myricanol [71]. The intramolecular cross Suzuki–Miyaura coupling of compound **67**, possessing an arylboronic acid pinacol ester and an aryl iodide resulted in a benzyl protected compound **68** as a racemic mixture. Debenzylation by hydrogenolysis, followed by enantioselective reduction of the keto group using K-selectride, led to a racemic mixture of **12aa**, which was then resolved by chiral high-performance liquid chromatography (HPLC) to afford (+)-**12aa**-(a*R*)(11*S*) and (−)-**12aa**′-(a*S*)(11*R*), of which the absolute configuration were determined by X-ray analysis. The starting compound **67** was prepared by Claisen-Schmidt reaction of **65** and **66** in 39% yield.


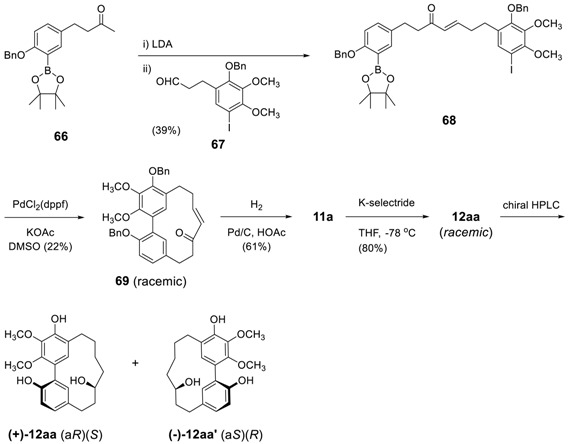


The emerging interest in (−)-a*S*,11*R*-myricanol owing to its ability to lower the tau protein level led to a couple of the enantioselective total synthesis: one covers a synthesis of the biaryl macrocycle skeleton via Suzuki–Miyaura cross coupling and ring-closing metathesis reactions [156], and the other includes an enantioselective asymmetric Suzuki cross-coupling, and an indium-mediated allylation of an aliphatic aldehyde [157]. Unfortunately, detailed procedures for these methods are currently unavailable. However, the successful enantioselective Suzuki–Miyaura cross coupling for synthesis of subclass linear diarylheptanoid, diospongin B [158], may open a vista to the enantioselective synthesis of biphenyl heptanoids.

#### 3.2.2. Aryl–Aryl Bond Formation via Photochemical Cyclization

Whiting and Wood employed photo-induced radical cyclization for the synthesis of myricanol [27,151]. The irradiation of the bromide **70** in EtOH in the presence of NaOH with a 252 nm wavelength light for 30 min afforded **72** in 10%, which could be readily converted to myricanol. However, an additional heptanoid **73**, expected to be formed via C,O-coupling in **71**, was not observed. The starting compound **70** were prepared from **52** by the method described for **53a** [152].


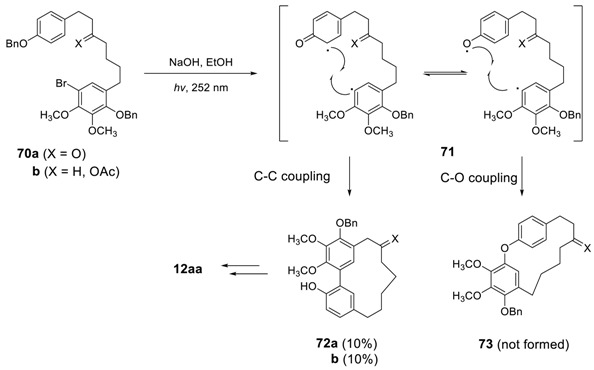


### 3.3. Total Synthesis of Cyclic Diphenyl Ether Heptanoids

The formation of diphenyl ether has long been challenging especially in the chemistry of polypeptide macrocycles and, therefore, led to the development of a couple of practical methods, such as oxidative coupling of phenols [159], the S_N_Ar reaction [160], the Ullmann reaction [161,162], and others [163,164].

#### 3.3.1. Formation of Macrocycles via Oxidative Coupling

The first attempt for the synthesis of diphenyl ether heptanoid comes from the biomimetic oxidative coupling of diarylheptanoid **76b** with Tl(OCOCF_3_)_3_ to yield a trace amount of the corresponding diarylheptanoid **77** [27]. Reactions with K_3_Fe(CN)_6_, Ag_2_O, MnO_2_ and VOCl_3_ produced only tars and thus no further applications were reported. Under these conditions, only C,O-coupling was proceeded to lead diphenyl ether heptanoid **77** unlike the result previously described above for **72a**. The prerequisite **76a** was prepared via Grignard reaction of 4-(2-benzyloxy-3,4-dimethoxyphenyl)butyl bromide (**51**) with 3-(4-benzyloxyphenyl)propanal (**74**), followed by hydrogenolysis.


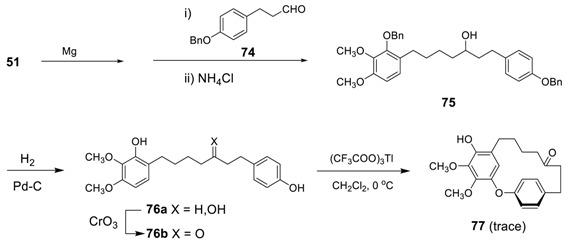


Recently, Salih and Beaudry examined various oxidative cyclization conditions for acerogenin G (**81**) [165] and identified the classical phenoxy radical-forming agent PbO_2_ in HOAc [166] as the oxidant of choice. Oxidative coupling of **81** with PbO_2_ in HOAc, thus, resulted in cyclization with concomitant oxidative hydroxylation of the diphenyl ether and with esterification of a resident phenol, leading to acetyl pterocarine (**82a**) and its regioisomer (**82b**) in a ratio of approximately 3:1. Interestingly, the reaction was completely chemoselective and this no oxidative C–C coupling product (i.e., acerogenin E) was observed. Although the yield was not somewhat low, one promising result is that 40% of the starting acerogenin G (**81**) was recoverable. The subsequent hydrolysis of **70a** afforded pterocarine (**26b**). The Horner–Wadsworth–Emmons reaction of phosphonate **78** with aldehyde **79** gave diene **80** in 88% yield, which was then subjected to catalytic hydrogenation to lead to starting acerogenin G (**81)** in 96% yield.


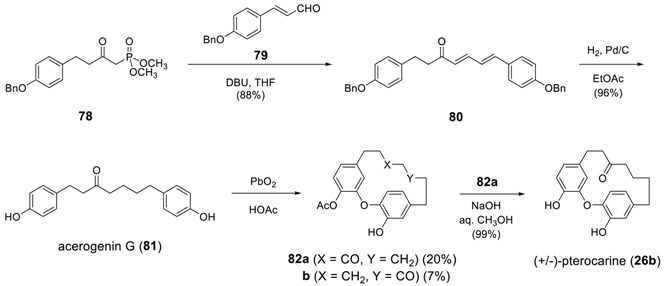


#### 3.3.2. Formation of Macrocycles via S_N_Ar Reaction

The first successful synthesis of acerogenin C (**25b**) included an intramolecular nucleophilic aromatic substitution (S_N_Ar) macrocyclization of **88a** to yield **89** [167]. In order to undertake such an S_N_Ar reaction efficiently, one or more strong electron withdrawing groups, such as a nitro group, are required at the proper positions [168]. The removal of the nitro group after the cyclization requires a couple of additional steps: the reduction of nitro to amine, followed by Doyle’s one-step deamination [169] to provide *O*-methylacerogenin C (**90a**). AlCl_3_ catalyzed demethylation of **90a** afforded acerogenin C (**25b**). The reaction of acerogenin C (**25b**) with 2,3,4,6-tetrabenzoylglucopyranosyl bromide in the presence of (*n*-Bu)_4_NBr provided glycoside **91** with 93% yield, of which subsequent saponification resulted in aceroside IV (**25a**) in 95% yield. The starting linear heptanoid **88a** was prepared by employing methyl acetoacetate ester synthesis. Dicarbanion generated from methyl acetoacetate (**83**) by LDA (2.1 equiv) was alkylated with **84** to give **85**, which was then subjected to 2nd alkylation with **86** in presence of NaH (1 equiv.) to provide **87**. Deprotection of the isopropyl group of **87**, followed by decarboxylation under acidic conditions, resulted in a linear diarylheptanoid **88**.


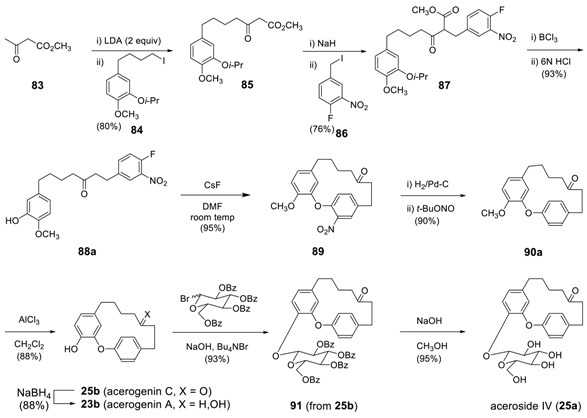


Such a strategy was later employed for the synthesis of acerogenins B and L by using **71b** as a starting material [170], which was similarly prepared from **92**.


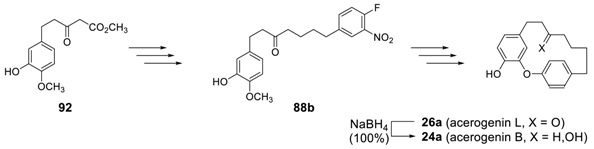


#### 3.3.3. Formation of Macrocycles via Intramolecular Ullmann Ether Synthesis

Keserü et al. has reported a synthesis of acerogenin A and C via an intramolecular Ullmann ether synthesis [171]. The linear diarylheptanoid **95** was heated at 130 °C in the presence of CuBr-Me_2_S and *t*-BuOK to afford the corresponding *O*-methylacerogenin C (**90a**) in 16% yield, which was then converted to acerogenin C (**25b**) via demethylation by pyridinium chloride. The subsequent reduction of **25b** by NaBH_4_ led to acerogenin A (**23b**). The prerequisite starting compound **95** was prepared by Wittig reaction of **93** with 4-iodobenzaldehyde and the subsequent catalytic hydrogenation of the double bond in **94**.


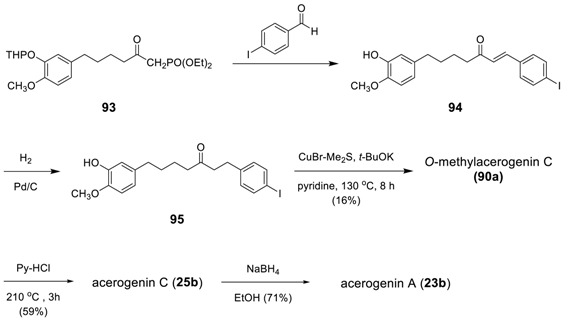


**Scheme 2:**
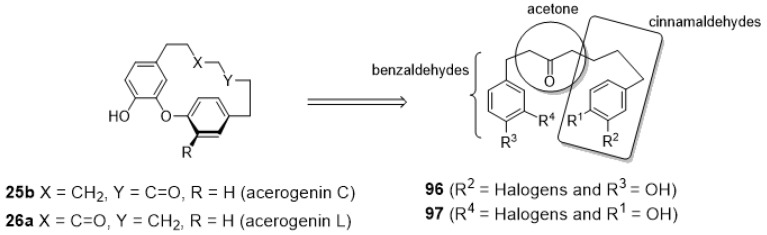
Retrosynthetic analysis for the synthesis of acerogenins.

Jahng and his coworkers [172] employed the same methodology for the synthesis of a series of acerogenins; the linear diarylheptanoids were prepared by employing a series of cross aldol condensations. Retrosynthetic analysis of the methods shown in Scheme 2 revealed that such a method holds the advantage that two types of acerogenins such as acerogenin C and L, can be prepared by the same reaction sequence via starting compounds of 4-halo-3-hydroxybenzaldehydes (**96** series) and 3-halo-4-hydroxybenzaldehydes (**97** series), respectively.

The Claisen–Schmidt condensation of **98a** with 4-benzyloxybenzaldehyde gave diarylheptenoid **99a**, which was then subjected to catalytic hydrogenation to yield **96a**. In the classical Ullmann reaction condition, CuO-K_2_CO_3_ in pyridine was applied to diarylheptanoid **96a** to yield *O*-methylacerogenin C (**90a**), from which the cleavage of methyl by treating AlCl_3_ afforded acerogenin C (**25b**). It is noteworthy that the application of 50 psi H_2_ to **99b** in the presence of 10% Pd/C resulted in the reduction of a double bond, the hydrogenolysis of benzyl ether as well as the Ar–Br bond. However catalytic hydrogenation at 1 atm H_2_ for 10 h led to the reduction of a double bond and the hydrogenolysis of benzyl ether without cleavage of the Ar–Br bond. The subsequent intramolecular Ullmann reaction cyclization reaction followed by demethylation afforded acerogenin L (**26a)**.


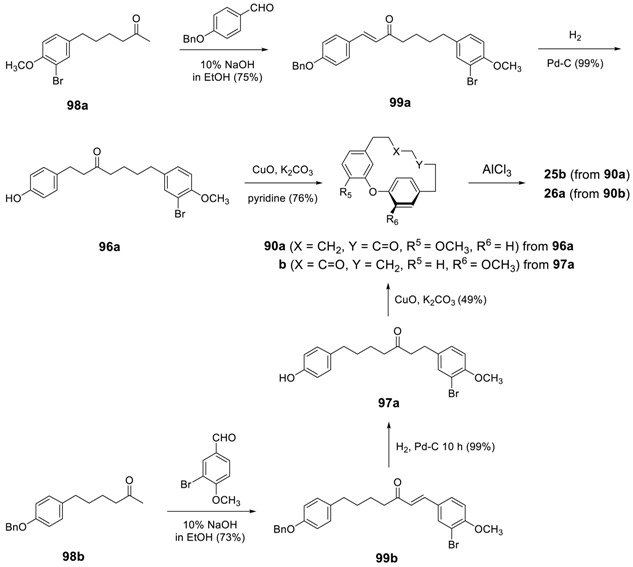


The same reaction sequence was applied to the synthesis of galeon and pterocarine [173]. The catalytic reduction of the double bond of **99c** afforded **97b**. The hydrogenation reaction time is critical for keeping the benzyl group: it can be minimized by stopping the reaction in 2.5 h at room temperature. The intramolecular Ullmann reaction of **97b** with CuO/K_2_CO_3_ in pyridine afforded **90c**, from which hydrogenolysis gave galeon (**26c**) and through subsequent demethylation of **26c** afforded pterocarine (**26d**). The starting **99c** was prepared by the same synthetic sequence employed above for **99a**,**b** by Claisen–Schmidt condensation of 6-(4-hydroxy-3-methoxyphenyl)hexan-2-one with 4-benzyloxy-3-bromobenzaldehyde.


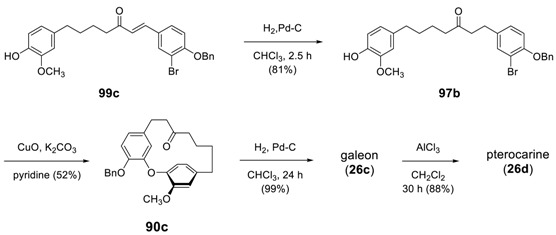


The same methodology was applied to **100a** to afford **101**, which gave the corresponding didemethylated product (**28**) in 42% yield by AlCl_3_-mediated cleavage of methyl ether moiety [174]. The ^1^H NMR spectrum of the product is not identical to that of engelhardione reported previously [120] and but did match that of pterocarine (vide ante). Based on these data, Shen and Sun revised the structure of engelhardione to be pterocarine [174] (vide ante). The starting **100a** was prepared from 6-(3-hydroxy-4-methoxyphenyl)hexan-2-one and 3-bromo-4-methoxybernzaldehyde via Claisen–Schmidt condensation followed by catalytic hydrogenation.


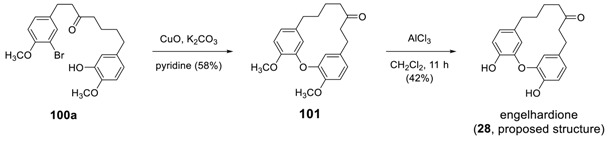


Recently, Salih and Beaudry reported the synthesis of a series of diphenyl ether-type diarylheptanoids such as myricatomentogenin (**26e**) and jugcathanin (juglanin A, **26f**) [175]. The Horner–Wadsworth–Emmons reaction of **102** with phosphonate **103** yielded hept-3,5-dien-2-ones **104**. The catalytic hydrogenation of **104a** and **104b**, followed by cyclization under Ullmann reaction conditions provided cyclophanes **90d** and **90e**, respectively. The selective cleavage of the isopropyl ether using BCl_3_ completed the synthesis of myricatomentogenin (**26e**) and jugcathanin (**26f**).


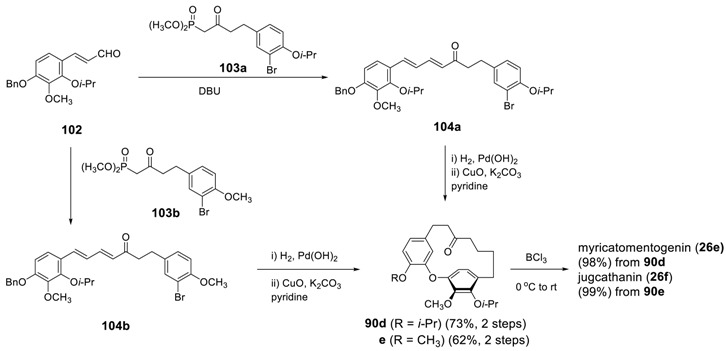


The starting cinnamaldehyde **102** was prepared in 4 steps from the corresponding coumarins via methanolysis, isopropyl ether formation, diisobutylaluminum hydride reduction and Dess–Martin oxidation.

The last part of diphenyl ether-type heptanoids describes the synthesis of garuganins and garugamblins. Beaudry and his coworkers reported an elegant synthesis and conformational dynamics of these series, in which they employed 1,7-diarylhept-3,5-diones as key intermediates [128,129]. To the lithium enolate of **105** was added an aldehyde **106** to produce an aldol product **107**. The oxidation of β-hydroxy ketone using IBX gave 1,3-diketone **108**, which was subjected to selective debenzylation by BCl_3_ at -78 °C to give **109**. An intramolecular Ullmann reaction using stoichiometric CuO in pyridine at the elevated temperature gave the cyclized product **110** in 38% yield. Methyl ether was achieved by treating CH_3_OH in presence of *p*-TsOH. In addition, the treatment of **28b** with CH_3_I afforded garuganin VI (**31**). Most of garuganins and garugamblins were prepared by using dihydrocinnamaldehyde derivatives **106** with appropriate substituents.


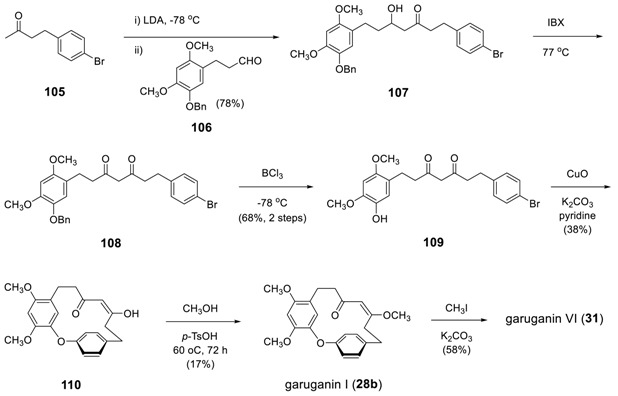


Finally tedarene A (**41a**) was prepared by the dehydration of **116**, which was prepared 4 steps from 3-(4-methoxyphenyl)propanal (**111**) and (4-(but-3-yn-1-yl)phenoxy)(*t*-butyl)dimethylsilane (**112**) [6] via Ullmann cyclization as a key reaction. An acetylide generated from **112** was reacted with **113** to afford the expected propargyl alcohol **113**. The optimized, controlled (6 atm of H_2_ for 18 h), and stereospecific hydrogenation of alkyne in **113** produced a *Z*-alkene (**114a**), which was then subjected to deprotection of the *t*-butyldimethylsilyl (TBS) group by tetra-*n*-butylammonium fluoroborate (TBAF) to give **114b**. The intramolecular Ullmann reaction of **114b**, followed by demethylation with NaSEt in refluxing DMF, afforded **115**. Kozikowski’s dehydration method using methanesulfonyl chloride in the presence of NEt_3_ in CH_2_Cl_2_ from 0 °C to room temperature [176] was applied to **115** to allow the elimination reaction to proceed, along with the unavoidable mesylation of the phenol leading to a mixture of diene *E*,*Z*- and *E*,*E*-isomers **116a** and **116b** in a 6:4 ratio (as determined by ^1^H NMR) in 80% yield. Hydrolysis of the mesylates was finally accomplished by aq. NaOH in an optimized 1:1 CH_3_OH/dioxane mixture at 60 °C, in 67% yield. Preparative HPLC chromatography using a chiral-phase column in direct phase allowed the separation of the two macrocycles, tedarene A (**41a**) and its isomer **41b** in 38.5% and 23% isolated yields, respectively, of which the structures were confirmed by X-ray structure analysis. The starting compound **111** was prepared in 2 steps from 3-(4-benzyloxyphenyl)propanal by regioselective bromination on the aromatic ring by Br_2_ in presence of AlCl_3_, and the subsequent IBX oxidation of the resulting alcohol led to the aldehyde.


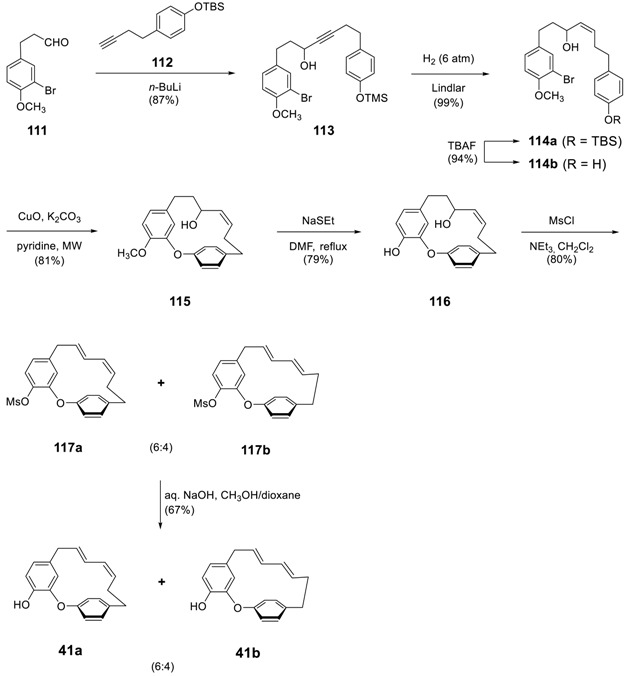


#### 3.3.4. Formation of Macrocycle via Formation of Heptane Skeleton

##### Via Wurtz and/or Wittig Reactions

The Wurtz and Wittig reactions were employed for the synthesis of garugamblin I, II, and III. Vermes et al. reported a synthesis of garugamblin I [177]: The Wittig reaction of the biaryl aldehyde **118** with **119** in the presence of *t*-BuOK afforded the alkene compound **120,** which was then converted to the dibromide compound **121** by a three-step reaction sequence. The radical anion induced by intramolecular Wurtz reaction with synchronistical cleavage of isoxazole provided β-enaminoketone **122** in 16% yield, where the intramolecular hydrogen bonding may force to adapt (Z)-alkene. The subsequent hydrolysis followed by methylation of enol with diazomethane led to two regioisomeric (*Z*)-enol ethers **123a** and **123b** Compound **123a** slowly isomerized to natural garugamblin I (**28a**) by simply standing in a chloroform solution for 2 weeks. The same isomerization has also been observed for **123b** to **124**. These observation may imply that *(Z)*-isomers [(*E*)-heptenes, **28a** and **124**] are somewhat more stable than the corresponding *(E)*-isomers [(*Z*)-heptenes, **123a** and **123b**, respectively.


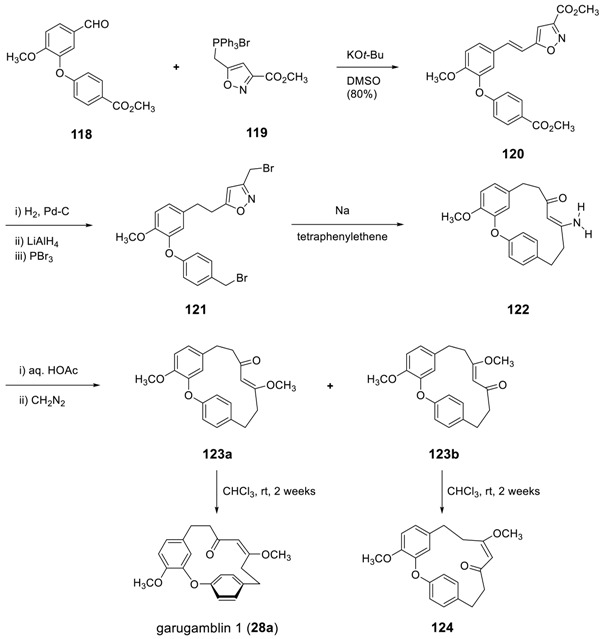


On the other hand, the same group employed a Wittig reaction for the synthesis of garuganin III (**32**) [128]. The intramolecular Wittig reaction was pursued by the addition of *t*-BuOK to a dilute solution of **125** in DMF to produce macrocycle **126**. The catalytic hydrogenation over PtO_2_ doped with Raney nickel saturated the double bond and cleaved isoxazole ring to give the enaminoketone **127**. The hydrolysis of **127** quantitatively resulted in the corresponding ketoenol, which was then methylated with diazomethane to yield garuganin III (**32**) with a mixture of its region- and stereoisomers. Based on the synthesis, the originally proposed structure was revised as shown. The same group employed identical synthetic methodology for the synthesis of garugamblin-2 (**31**) [178].


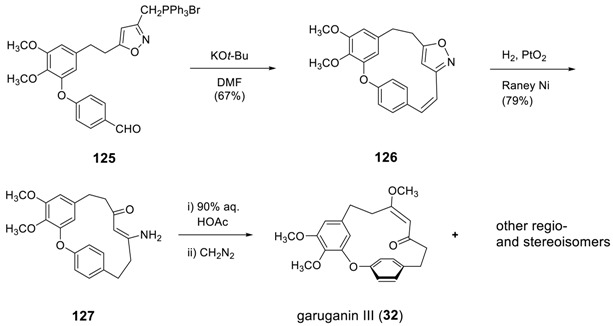


##### Via Ring-Closing Metathesis

Ring-closing metathesis has been used as a powerful tool for macrocyclization [147]. The intermolecular Ullmann reaction of **128** and **129** afforded the prerequisite precursor **130**, of which the ring closure metathesis with a variety of Grubbs’ catalysts for the synthesis of ovalifoliolatin B (**35b**) [179] did not proceed at all. On the other hand, it is worth noting that an intermolecular ring closure metathesis of **128** and **129** led to an inseparable mixture of stereoisomers of the precursor **131**, which was then CuO mediated Ullmann cyclization to provide ovalifoliolatin B (**35b**) and its *cis*-isomer **132** in a ratio of 13:1. The catalytic hydrogenation of these two isomers, followed by demethylation by AlCl_3_ afforded acerogenin C (**25b**). All attempts for the synthesis of cyclic diarylheptanoids employing ring closing metathesis have, thus far, failed implying the scope of such a reaction for the application towards the synthesis of cyclic diaryl ether heptanoids.


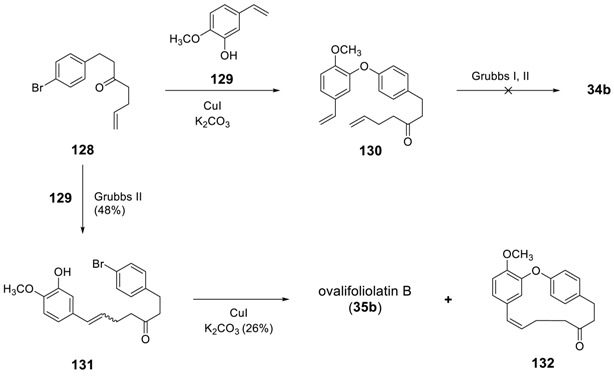


#### 3.3.5. Enantioselective Synthesis of Diaryl Ether Heptanoids

Two types of enantioselective reactions, such as enantioselective Ullmann ether coupling and chiral phase-transfer-catalyzed atropselective diaryl ether formation via S_N_Ar reaction, have been reported.

Salih and Beaudry reported the first asymmetric syntheses of (−)-myricatomentogenin, (−)-jugcathanin, (+)-galeon, and (+)-pterocarine by enantioselective Ullmann cross-coupling reaction [180]. The intramolecular coupling reaction was evaluated using enantiopure ligands known to accelerate the Ullmann reaction and some other privileged ligand structures. Cu-catalyzed cross coupling of **97c** in the presence of BINOL-type ligands would lead enantioselectivity with low chemical yields. Among the 21 tested ligands, *N*-methyl-l-proline did lead to not only increased chemical yield up to 41% but also enantioselectivity up to 68:32 in the presence of 20 mol% CuI. Variations in the *N*-alkyl group, the ring size, and the carboxylic acid functionality did not significantly improve the yield or enantioselectivity of the reaction. The survey of a variety of inorganic and organic bases in the Ullmann coupling indicated that use of K_3_PO_4_ instead of Cs_2_CO_3_ could be the conditions of choice with a higher enantiomeric ration without a significant loss of chemical yield (see Table 7). Thus, enantioselective cross Ullmann coupling of **97c** was pursued under the optimized reaction conditions [CuI (20 mol %), *N*-methyl-l-proline (40 mol %), K_3_PO_4_ (2 equiv.) in dioxane] afforded **(p*R*)-90f** with 72:28 er. Although they did not find any better system, such enantiomeric ratio can be improved to 92:8 er by recrystallization. The partial and complete demethylation of **(p*R*)-90f** afforded (+)-galeon (43%, (92:8 er) and (+)-pterocarine (45%, 92:8), respectively.


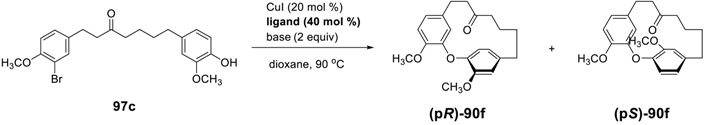


Subsequent Ullmann cross-coupling of **97d** under optimized conditions [CuI (20% mol) in the presence of K_3_PO_4_ (2 equiv) and chiral ligand, *N*-methyl-l-proline (40 mol%)] afforded **90g** in moderate yield and with a degree of enantioselectivity (67:33 er). The enantiomeric ratio of cyclophane **90g** was increased up to 82:18 er by additional recrystallization. The treatment of **90g** with BCl_3_ gave a 1:1 mixture of the product with one isopropyl group (**90h**) and (−)-myricatomentogenin (**26e**) in nearly quantitative yield without any loss in enantioenrichment. Subsequent methylation followed by the removal of isopropyl group in **90h** provided (−)-jugcathanin (**26f**) without any loss in enantiomeric ratio.


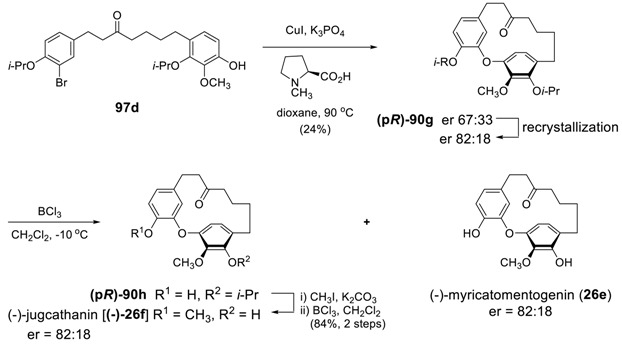


Alternatively, Ding et al. used the chiral phase transfer-transfer catalyst to induce an asymmetry during intramolecular S_N_Ar cyclization for the formation of diphenyl ether [181]. They screened the reaction conditions in the presence of various chiral phase-transfer catalysts and found the cinchonine-derived ligand **133** as a phase-transfer catalyst of choice and 20% CsOH in toluene as base and solvent (see Table 8) as a best reaction condition.

Therefore, the intramolecular S_N_Ar reaction of **88b** was explored on a 1 g scale in the presence of PTC **133** (20 mol%), 20% CsOH (aq) in toluene at room temperature to afford the desired product **(p*S*)-89** in 81% yield with an er of 91:9. This er was improved up to 99:1 by recrystallization, with overall 62% yield. In addition, the hydrogenation of **(p*S*)-89** provided the corresponding amino compound **134**, of which the absolute configuration was determined as p*S* by X-ray crystallography. Compound **134** was further converted to a hydroxyl compound **135** without any measurable loss of enantiopurity by using a previously published method [170]. The demethylation of **135** led to pterocarine, which was determined to be (−)-pterocarine by comparison with previously reported data for its enantiomer [25].


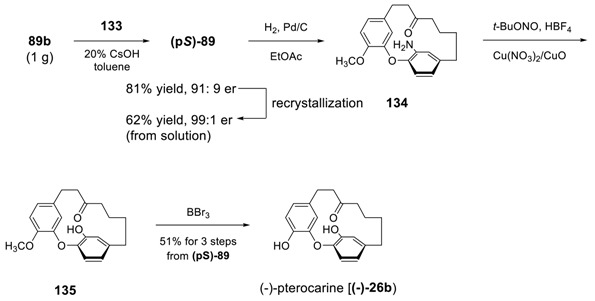


Similarly, the same reaction sequence starting with counter precursor (**88c**) of **88a** afforded the desired compound **136** in 83% yield, but with only moderate enantioselectivity (69:31 er). However the highly (99:1 er) (−)-enriched **136** could be obtained by simple recrystallization, which then converted to (−)-galeon by a simple transformation procedure reported previously.


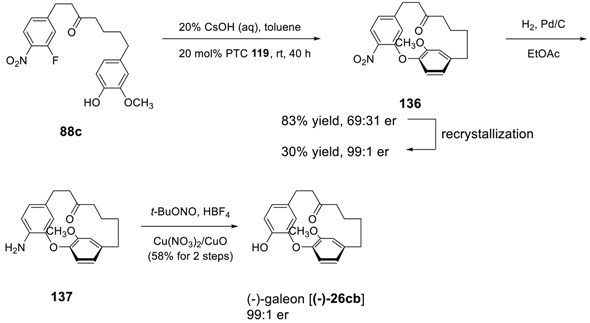


## 4. Conclusion

Steady progress in the chemistry and biology of cyclic diarylheptanoids has resulted in a diverse range of new structures and new biological activity profiles. Slow but continuous efforts on the synthesis of cyclic diarylheptanoids have led not only to the revision of originally proposed structures, but also to the identification of synthetic methods to establish atropenantiomers. Partial success in the enantioselective synthesis of (−)-myricatomentogenin, (−)-jugcathanin, (+)-galeon, and (+)-pterocarine by Ullmann cross coupling in the presence of chiral ligands and enantioselective synthesis of (−)-pterocarine and (−)-galeon by enantioselective S_N_Ar formation in the presence of chiral phase-transfer catalyst may open a door to the development of an enantioselective macrocyclization for diphenyl ether heptanoids. On the other hand, a couple of the enantioselective total syntheses for the biaryl macrocycle skeleton via Suzuki–Miyaura cross-coupling and successful enantioselective Suzuki–Miyaura cross-coupling for the synthesis of subclass linear diarylheptanoid, diospongin B [158] may lead to a practical enantioselective synthesis of biphenyl heptanoids.

However, to the best of our knowledge, the structure–activity relationship (SAR) study of cyclic diarylheptanoids is very limited [80,182,183]; thus, the identification of good drug candidates has not been fruitful yet. Not only development of a facile enantioselective macrocyclization method but also more intense SAR study have become a prerequisite for realizing such a goal.

## Figures and Tables

**Table 1:** Occurrence of diarylheptanoids in plants reported in the literature.

Family	Genus	Diarylheptanoids			Reference(s)
		Linear (Type I)	Biphenyl (Type II)	Diphenyl Ether (Type III)	
Aceraceae	*Acer*	+	+	+	[7]
Actinidiaceae	*Clematoclethra*	−	−	+	[8]
Betulaceae	*Alnus*	+	+	+	[9]
	*Betula*	+	+	+	[10]
	*Corylus*	−	+	−	[11,12]
	*Carpinus*	−	+	−	[13]
	*Ostrya*	−	+	−	[14]
	*Ostryopsis*	+	+	+	[15,16]
Burseraceae	*Garuga*	−	+	+	[17,18]
	*Boswellia*	−	−	+	[19]
Casuarinaceae	*Casuarina*	−	+	−	[20]
Juglandaceae	*Engelhardia*	+	−	+	[21]
	*Juglans*	+	+	+	[22,23]
	*Platycarya*	−	+	+	[24]
	*Pterocarya*	−	−	+	[25]
	*Rhoiptelea*	+	+	+	[26]
Myricaceae	*Myrica*	−	+	+	[27,28]
	*Morella*	+	+	+	[29]
Rubiaceae	*Scyphiphora*	−	+	−	[30]
Zingiberaceae	*Alpinia*	+	−	−	[31]
	*Curcuma*	+	−	−	[4]

**Table 2:**
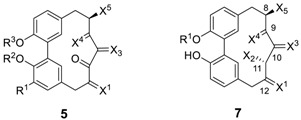
Derivatives of alnusonol (**5a**) and alnusdiol (**7a**), and their stereochemistry.

	R^1^	R^2^	R^3^	X^1^	X^2^	X^3^	X^4^	X^5^	Chiral Axis	Reference and Biological Activity
alnusonol (**5a**)	H	H	H	H,H	-	H,H	H,OH (*S*)	H		α-glucosidase inhibitor [65]
alnusdiol (**7a**)	H	H	H	H,H	OH (*S*)	H,H	H,OH (*S*)	H	a*S*	[51]
**5c**	H	H	H	H,H	-	H,H	H,OH	OH		[53]
**5d**	H	H	H	H,H	-	H,OH	O	H		anti-adipogenic activity [55]
giffonin L ^a^ (**7d**)	H	H	H	H,OH (*S*)	OH (*S*)	H,OH (*R*)	H,H	OH (*S*)		[12]
giffonin M (**5e**)	H	H	H	H,H	-	H,OH (*S*)	H,H	OH (*S*)		[12]
giffonin N (**5f**)	H	H	H	H,H	-	H,OH (*S*)	H,H	*O*-β-d-Glc (*S*)		[12]
giffonin O (**7e**)	H	H	H	H,OH (*R*)	H	O	H,OH (*R*)	OH (*S*)		[12]
giffonin P ^a^ (**7f**)	H	H	H	H,OH (*R*)	OH (*S*)	H,OH	H,OH (*S*)	OH (*S*)		[12]
giffonin T (**5g**)	H	R′ ^b^	H	H,H	-	H,OH (*R*)	H,OH (*S*)	OH (*R*)	a*S*	[64]
giffonin U (**7g**)	H	H	H	O	OH (*R*)	H,OH (*R*)	H,OH (*R*)	OH (*R*)	a*S*	[64]
carpinontriol A (**5h**)	H	H	H	H,OH (*R*)	-	H,H	H,OH (*S*)	OH (*R*)		[13]
carpinontriol B (**5i**)	H	H	H	H,H	-	H,OH (*R*)	H,OH (*S*)	OH (*R*)	a*S*	[13], inhibit lipid peroxidation [64]
**5j**	OH	CH_3_	H	H,H	-	H,H	H,H	H		[56], leishmanicidal (IC_50_ = 17 mg/mL) [63]
aceroside XI ^c^ (**5k**)	H	H	R′ ^b^	H,H	-	H,H	H,H	H		[66]
acerogenin E ^d^ (**5l**)	H	H	H	H,H	-	H,H	H,H	H		NO production inhibitor (IC_50_ = 24 μM) [67]
acerogenin K (**7h**)	H	H	H	H,H	OH	H,H	H,H	H		NO production inhibitor (IC_50_ = 24 μM) [67]
ostryopsitriol (**7i**)	H	H	H	H,OH	OH	H,H	H,OH	H		[16]
betulatetraol (**7j**)	H	H	H	H,OH	OH	H,H	H,OH	OH		[43]

^a^ Giffonin A–K, Q, R, and S are diphenyl ether heptanoids (*vide infra*). ^b^ R’ = β-d-glucopyranosyl. ^c^ Aceroside I–X are cyclic diphenyl ether heptanoids (*vide infra*). ^d^ Acerogenins A–D, F, H–J, L, and M are cyclic diphenyl ether heptanoids (*vide infra*).

**Table 3:**
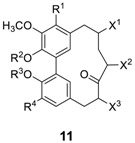
The derivatives of myricanone (**11a**) and their biological properties.

Compound	R^1^	R^2^	R^3^	R^4^	X^1^	X^2^	X^3^	Reference(s) and Biological Properties
**11a** (myricanone)	OH	CH_3_	H	H	H	H	H	cytotoxicity [76,77], cell apoptosis [78], anti-inflammatory in iNOS assay (IC_50_ =1.0 μM) [79], radical scavenging (IC_50_ = 19.6 μM) [79], lowering protein tau level [71], chemo-preventive ^a^ (IC_50_ = 320) [80]
**11b**	*O*-β-Glc	CH_3_	H	H	H	H	H	DPPH radical scavenging activity (49.09%) [81]
**11c** (alnuheptanoid B)	*O*-β-Glc	CH_3_	COCH_3_	H	H	H	H	DPPH radical scavenging activity (41.16%) [81]
**11d** (saliciclaireone A)	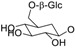	CH_3_	H	H	H	H	H	[29]
**11e** (saliciclaireone C)	OCH_3_	H	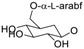	H	H	H	H	[29]
**11f**	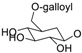	CH_3_	H	H	H	H	H	[29]
myricananin C (**11g**)	H	H	H	H	H	H	H	NO release inhibition (IC_50_ = 64.51 μM) [82]
myricananin E (**11h**)	OH	CH_3_	H	OH	H	H	OCH_3_	[82]
myricananone (**11i**)	OH	CH_3_	H	H	OH	H	H	[83]
**11j**	H	CH_3_	H	H	H	H	H	[84], anti-tubercular activity (IC_50_ = 25.8 μg/mL) [79]
**11k**	OH	CH_3_	H	H	H	H	OH	anti-tubercular activity (IC_50_ = 35.8 μg/mL) [79]

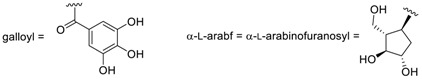

^a^ Value is the concentration (Mol ratio/TPA) needed to inhibit 50% of positive control activated with TPA.

**Table 4:**
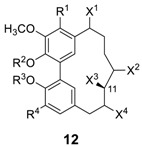
The derivatives of myricanol (**12a**) and their biological properties.

Compound	R^1^	R^2^	R^3^	R^4^	X^1^	X^2^	X^3^	X^4^	Reference(s) and Biological Properties
myricanol (**12a**)	OH	CH_3_	H	H	H	H	OH (*S*)	H	[68], radical scavenging (IC_50_ = 22.3 μM) [79], anti-Alzheimer’s disease by lowering tau level [71]
juglanin B (**12ba**)	H	H	H	H	H	H	OH (*S*)	H	cytotoxic (HT-29) [40]
salicireneol A (**12c**)	H	β-Glc	H	H	H	H	OH (*S*)	H	[29]
salicireneol B (**12d**)	H	H	β-Glc	OH	H	H	OH (*S*)	H	[29]
myricananin A (**12e**)	H	H	H	H	H	H	OH (*S*)	OH (*S*)	NO release inhibitor (IC^50^ = 45.32 μM) [82]
myricananin B (**12f**)	OH	CH_3_	H	H	H	H	OH (*S*)	OH (*S*)	[82]
myricananin F (**12g**)	H	H	H	H	H	OH	H	H	[85]
myricananin G (**12h**)	OH	CH_3_	CH_3_	H	H	H	OH (*S*)	OH (*R*)	[85]
myricananin H (**12i**)	OCH_3_	CH_3_	CH_3_	H	H	H	OH (*S*)	OH (*S*)	[85]
salicimeckol (**12j**)	*O*-β-Glc	CH_3_	H	H	OH	H	OH (*S*)	H	[29]
**12k**	*O*-β-Glc	CH_3_	H	H	H	H	OH (*S*)	H	anti-allergic activity [86]
**12l**	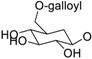	CH_3_	H	H	H	H	OH (*S*)	H	[87], DPPH radical scavenging (IC_50_ = 6.8 μM) [88], SOD-like activity (IC_50_ = 90.5 μg/mL) [89]
**12m**	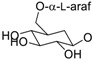	CH_3_	H	H	H	H	OH (*S*)		[90]
**12n**	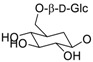	CH_3_	H	H	H	H	OH (*S*)		[87]

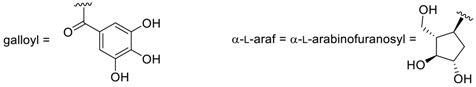

**Table 5:**
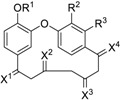
The acerogenins with diphenyl ether heptanoid skeleton and their biological properties.

Compound	R^1^	R^2^	R^3^	X^1^	X^2^	X^3^	X^4^	Reference and Biological Properties
acerogenin A (**23b**)	H	H	H	H,H	H,H	H,OH (*R*)	H,H	anti-inflammatory (IC_50_ = 0.32 mg/ear) [107], osteogenic activity [108]
acerogenin B (**24a**)	H	H	H	H,H	H,OH	H,H	H,H	cytotoxicity (IC_50_ = 25.1 μM) of (*R*)-isomer against HL60 [109], Na^+^-glucose cotransporter inhibitor [105]
acerogenin C (**25b**)	H	H	H	H,H	H,H	O	H,H	antibacterial and neuroprotective [110], NO production inhibitor (IC_50_ = 61.4 μM) [111]
acerogenin D (**25c**)	H	H	H	H,H	H,OH	O	H,H	radical scavenging activity (IC_50_ = 40 μM) [62]
acerogenin F (**23e**) ^a^	H	H	H	H,H	H,H	H,OH (*R*)	H,OH (*R*)	[60]
acerogenin H (**23f**)	H	H	H	O	H,H	H,OH	H,H	[64]
acerogenin I (**24b**)	H	H	H	H,H	H,OH	H,H	H,OH	[64]
acerogenin J (**23g**)	H	H	H	H,H	H,H	H,OH (*R*)	H,OH (*S*)	[64]
acerogenin L (**26a**)	H	H	H	H,H	O	H,H	H,H	[64]
acerogenin M (**24c**)	H	H	H	O	H,OH	H,H	H,H	antitumor promoting activity [107]
pterocarine (**26b**)	H	OH	H	H,H	O	H,H	H,H	cytotoxic against K562 (51% @100 μg/mL) [25]
galeon (**26c**)	H	OCH_3_	H	H,H	O	H,H	H,H	[104]
10-hydroxygaleon (**26d**)	H	OCH_3_	H	H,OH	O	H,H	H,H	[104]
**24d**	H	OCH_3_	H	H,H	H,OH (*R*)	H,H	H,H	[23]
myricatomentogenin (**26e**)	H	OCH_3_	OH	H,H	O	H,H	H,H	[112]
jugcathanin (juglanin A, **26f**)	CH_3_	OCH_3_	OH	H,H	O	H,H	H,H	cytotoxicity [40,91]
platycarynol (**24e**)	CH_3_	OCH_3_	H	H,H	H,OH (*R*)	H,H	H,H	[24], cytotoxic against A549 (IC_50_ = 11.5 μg/mL) [22], NF-kB inhibitor [113]
aceroside B_1_ (**24fa**)	β-d-Glc	H	H	H,H	H,OH (*R*)	H,H	H,H	[61], osteogenic activity [108]
aceroside B_2_ (**24fb**)	β-d-Glc	H	H	H,H	H,OH (*S*)	H,H	H,H	[61]
aceroside I (**23h**)	β-d-Glc	H	H	H,H	H,H	H,OH (*R*)	H,H	[114], osteogenic activity [108]
aceroside II (**24g**)	H	H	H	H,H	H, O-β-d-Glc	H,H	H,H	[60]
aceroside III (**23i**)	H	H	H	H,H	H,H	H,Y ^c^	H,H	radical scavenging activity (IC_50_ = 40 μM), osteogenic activity [108]
aceroside IV (**25a**)	β-d-Glc	H	H	H,H	H,H	O	H,H	[106]
aceroside V (**25d**)	β-d-Glc	H	H	H,H	H,OH	O	H,H	[62]
aceroside VI ^b^ (**23j**)	H	H	H	H,H	H,H	H,*O*-β-d-Glc	H,H	[115]
9-oxoacerogenin A (**23k**)	H	H	H	H,H	O	H,OH	H,H	anti-melanogenesis (17.6% @100 μM) [109]
maximowicziol A (**24h**)	H	H	H	H,H	H,OH (*S*)	H,H	H,OH (*S*)	[52]
**24i**	CH_3_	OCH_3_	OH	H,H	H,OH	H,H	H,H	cytotoxic against A549 (IC_50_ = 11.5 μg/mL) [22]
jugsigin A (**24j**)	H	H	OH	H,H	H,OH (*S*)	H,H	H,H	cytotoxic against HT-29 [116]
2-methylacerogenin A (**23l**)	CH_3_	H	H	H,H	H,H	H,OH (*S*)	H,H	[100]

^a^ Acerogenin E (**5l**) and K (**7h**) are biphenyl heptanoids, acerogenin G is a linear diarylheptanoid, 7-(3-hydroxyphenyl)-1-(4-hydroxyphenyl)heptan-3-one [117]. ^b^ At present, 11 acerosides have been reported: acerogenins VII and VIII are 3-*O*-β-d-glucopyranosides of linear diarylheptanoid, 1,7-di(4-hydroxyphenyl)heptan-3-ol [118], acerosides IX and X are β-d-glucopyranosides of linear diarylheptanoid, 1,7-di(4-hydroxyphenyl)heptan-3-one [117], and aceroside XI is **5k**. ^c^ Y = *O*-β-d-Api-(1->6)-β-d-Glc.

**Table 6:**
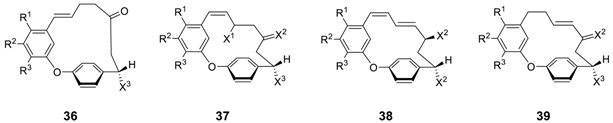
Diaryl ether heptenoids, giffonin A–K, Q, R, and S.

	R^1^	R^2^	R^3^	X^1^	X^2^	X^3^	Reference
giffonin A (**36a**)	OCH_3_	OH	OCH_3_	-	-	H	[11]
giffonin B (**36b**)	OCH_3_	OH	OCH_3_	-	-	OH (*S*)	[11]
giffonin C (**37a**)	OCH_3_	OH	OCH_3_	H	H,OH (*R*)	H	[11]
giffonin D (**37b**)	OCH_3_	OH	OCH_3_	H	O	H	[11]
giffonin E (**37c**)	OCH_3_	OH	OCH_3_	OH (*R*)	H,H	H	[11]
giffonin F (**37d**)	OCH_3_	OH	OCH_3_	OH (*R*)	O	H	[11]
giffonin G (**38a**)	OCH_3_	OH	OCH_3_	-	H,OH (*S*)	H	[11]
giffonin H (**38b**)	H	OH	OCH_3_	-	H,OH (*S*)	H	[11]
giffonin J ^a^ (**37e**)	OCH_3_	OH	OCH_3_	H	H,OH (*R*)	OH (*S*)	[12]
giffonin K (**38c**)	OCH_3_	OH	OCH_3_	-	H,OH (*S*)	OH (*S*)	[12]
giffonin Q (**39**)	H	H	H	-	O	H	[132]
giffonin R (**38d**)	H	H	OH	-	O	H	[132]
giffonin S (**38e**)	H	OCH_3_	OH	-	O	H	[132]

^a^ Giffonin I, L–P, T, and U are biphenyl heptanoids (*vide supra*).

**Table 7:** Optimization of reaction conditions.

Ligand	Base	Yield (%) ^a^	Ratio (p^R^ : p^S^) ^b^
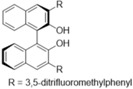	Cs_2_CO_3_	18	38: 62
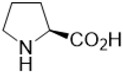	Cs_2_CO_3_	22	44: 56
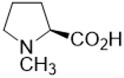	Cs_2_CO_3_	41	68: 32
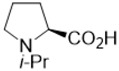	Cs_2_CO_3_	34	57: 43
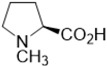	K_3_PO_4_	39	72: 28

^a^ Isolated yield (average of three trials). ^b^ Determined by HPLC.

**Table 8:**
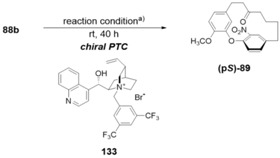
Screening reaction conditions for cyclization of linear diarylheptanoid **88b**.

Solvent	Base (aq. Solution)	Yield (%) ^b)^	Er ^c)^
toluene (5 mL)	20% CsOH	47	87: 13
DMF (5 mL)	20% CsOH	90	50: 50
xylene (5 mL)	20% CsOH	47	82: 18
toluene (5 mL)	20% CsF	<10	69: 31
toluene (5 mL)	20% KOH	17	64: 36
toluene (5 mL)	20% CsOH	46	89: 11
toluene (5 mL) ^d)^	20% CsOH	80	91: 9
toluene (5 mL) ^d)^	20% CsOH	47	91.5: 8.5

^a)^ Reagents and reaction conditions: **88b** (37.5 mg, 0.1 mmol), PTC (10 mol%), base (15 equiv), rt, 40 h. ^b)^ Isolated yields. ^c)^ Determined by high-performance liquid chromatography (HPLC) analysis. ^d)^ 20 mol% PTC used.
